# Reciprocal Subsidies and Food Web Pathways Leading to Chum Salmon Fry in a Temperate Marine-Terrestrial Ecotone

**DOI:** 10.1371/journal.pone.0010073

**Published:** 2010-04-08

**Authors:** Tamara N. Romanuk, Colin D. Levings

**Affiliations:** 1 Department of Biology, Dalhousie University, Halifax, Nova Scotia, Canada; 2 Centre for Aquaculture and Environmental Research, Department of Fisheries and Oceans, West Vancouver, British Columbia, Canada; University of Canterbury, New Zealand

## Abstract

Stable isotope analysis was used to determine the relative proportions of terrestrial and marine subsidies of carbon to invertebrates along a tidal gradient (low-intertidal, mid-intertidal, high-intertidal, supralittoral) and to determine the relative importance of terrestrial carbon in food web pathways leading to chum salmon fry *Oncorhynchus keta* (Walbaum) in Howe Sound, British Columbia. We found a clear gradient in the proportion of terrestrially derived carbon along the tidal gradient ranging from 68% across all invertebrate taxa in the supralittoral to 25% in the high-intertidal, 20% in the mid-intertidal, and 12% in the low-intertidal. Stable isotope values of chum salmon fry indicated carbon contributions from both terrestrial and marine sources, with terrestrially derived carbon ranging from 12.8 to 61.5% in the muscle tissue of chum salmon fry (mean 30%). Our results provide evidence for reciprocal subsidies of marine and terrestrially derived carbon on beaches in the estuary and suggest that the vegetated supralittoral is an important trophic link in supplying terrestrial carbon to nearshore food webs.

## Introduction

Subsidies of prey and detritus across ecotones have been shown to affect food webs in both aquatic and terrestrial habitats [1]–[3]. In coastal areas, nearshore marine habitats commonly receive prey and detritus from adjacent terrestrial habitats [2]. This transfer of nutrients from terrestrial to marine habitats is also reciprocal, with nutrients derived from the marine environment entering terrestrial habitats in the form of beach wrack [2].

Supralittoral vegetation in coastal areas may play similar roles in ecosystem functioning as riparian vegetation in freshwater systems [4]. In small watersheds with dense surrounding forests much of the stream organic matter originates in the surrounding forest [5] and in freshwater riparian and stream food webs terrestrial invertebrates can comprise more than 50% of energy intake by stream fishes and are often a preferred prey of salmonids [6]. Similarly, in marine coastal habitats, supralittoral vegetation may provide an important source of terrigenous input in the form of leaf litter to intertidal areas [7]–[8] and terrestrial and intertidal invertebrates have been shown to comprise a proportion of their diets of salmon fry caught in nearshore habitats [9]–[12], [14]–[15].

Marine sources of carbon and nitrogen have also been shown to subsidize terrestrial food webs [16]. Marine subsidies are particularly pronounced on islands, which often have extremely low terrestrial primary productivity [16]–[17] and for ecosystems with high throughputs of anadromous fishes such as salmonids, which subsidize terrestrial vegetation [18]–[19]. For example Hocking and Reimchen (2009) found that the δ ^15^N signatures of riparian vegetation in 27 watersheds in British Columbia was positively related to total the biomass of spawning chum and pink salmon [19].

On coastal beaches, beach wrack is an important food source and habitat that subsidizes both marine and terrestrial food webs. For example, Lewis et al. [20] have shown that beach wrack subsidizes marine shore crabs that ride the nightly tide to the wrack line to feed on talitrid amphipods which forage at night on the beach wrack. Wrack also provides food for terrestrial organisms, in particular terrestrial arthropods [17], [21]–[24]. Olabarria et al. [25] found that beach wrack arthropod communities were dominated by terrestrial consumers such as coleopteran tenebrionid and staphylinid species and dipteran flies.

Stable isotope analysis (SIA) has been used extensively to describe aquatic food webs [26] and has become increasingly popular method to quantify energy flow, especially in ecotones where the contributions of terrestrial and aquatic energy sources have distinct isotopic signatures [27]–[28]. The ratio of the stable isotopes of nitrogen ^15^N/^14^N is positively correlated with trophic level, and the ratio of carbon stable isotopes ^13^C/^12^C yields information about the production base of the food web [26]. Carbon fixed by terrestrial C3 plants in temperate regions has a characteristic ^13^C/^12^C ratio of approximately −28‰ [29]. Aquatic plants exhibit a much wider range in δ^13^C (−50‰ to −10‰) relative to terrestrial plants, reflecting site-specific and species-specific factors [30]–[31]. Because terrestrial and aquatic primary producers often have distinct carbon sources, mixing models can be used to assess the relative proportions of these primary energy sources in consumer diets [32].

In this study we report the results of stable isotope analysis of carbon and nitrogen for a collection of marine, intertidal, and terrestrial organisms collected in the intertidal and supralittoral in Howe Sound, British Columbia, Canada. Our objective was to determine the proportion of terrestrially derived carbon (TC) and marine derived carbon (MC) along the intertidal to supralittoral gradient focusing specifically on the pathways of energy flow to chum salmon fry, *Oncorhynchus keta* (Walbaum), which reside in the estuary from March to June during their transition to the marine environment.

## Methods

**Figure 1:**
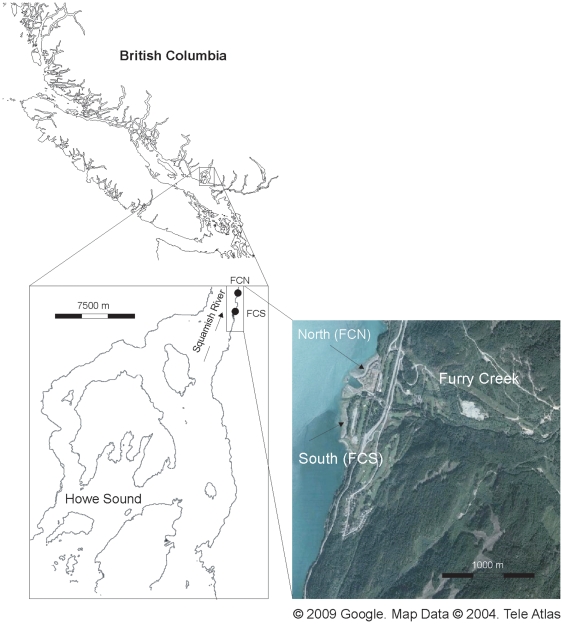
Map of the British Columbia, the Strait of Georgia, and Howe Sound showing the two beaches (Furry Creek, North and South: FCN, FCS). Aerial image of Furry Creek showing the location of the two beaches on either side of the creek (© 2009. Google. Map Data. 2004 Tele Atlas).

**Figure 2:**
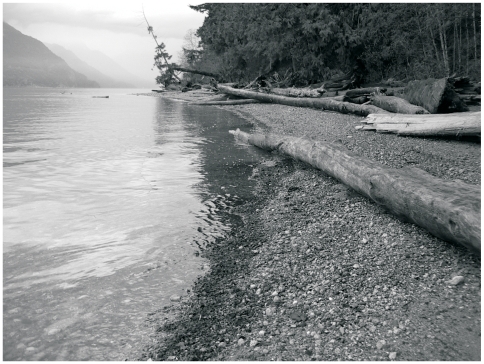
Beach at Furry Creek (South), Howe Sound, British Columbia at high tide showing wrack line and supralittoral vegetation.

Howe Sound is a fjord located on the southeastern shore of the Strait of Georgia, British Columbia, Canada (Fig. 1). The Sound derives its estuarine characteristics from the Squamish River on the northern reaches and the Fraser River on the southern reaches, as well as smaller creeks along the shoreline. Between March and October 2002 we collected samples of supralittoral vegetation, macroalgae, invertebrates, and chum salmon fry on two beaches at Furry Creek, located on the east side of the Sound (Fig. 1). The creek is located between the North and South sites. Several species of salmon (chinook, coho, chum, pink) have been found in Furry Creek but because major runs of chum salmon occur in the Squamish and Fraser Rivers it is probable that most of the chum fry we sampled were from the latter two river systems. At Furry Creek South, where there is >50 m swath of intact supralittoral vegetation we collected supralittoral vegetation, macroalgae, invertebrates, and chum salmon fry (Fig. 2). At Furry Creek North, where the supralittoral vegetation was removed for a housing development, we only collected chum salmon fry. For additional details regarding the sites see Romanuk and Levings [12]–[13]. The beaches are within ∼350 m of each other. Range of tidal heights during the sampling period was from 0.28 m to 4.85 m±1.29 SD.

Ten species of live terrestrial supralittoral plants and six species of live macroalgae were collected by hand at Furry Creek [12]. Samples of vegetation and algae were washed with distilled water and then frozen and stored. Invertebrates were collected in June and October in four distinct zones: supralittoral, high-intertidal (i.e. beach wrack zone), mid-intertidal, and low-intertidal zones. Sampling similar Orders across zones allowed us to compare how δ^13^C changed along the terrestrial to marine gradient. Three Orders were sampled in more than one zone: Diptera (primarily Chironomidae) were sampled in the supralittoral (adult), high-intertidal (adult), mid-intertidal (adult), and low-intertidal (larvae) zones; Acariformes were collected from the supralittoral, high- and mid-intertidal zone; Amphipoda (Talitridae) were collected from the high-, mid-, and low-intertidal zones. Gastropods and *Mytilus* sp. were collected in the mid-intertidal.

We used a variety of collection methods including epibenthic sleds in the low-intertidal zone and hand vacuums in the supralittoral, high-intertidal, and mid-intertidal zones. Taxa were identified to lowest taxonomic level possible while retaining enough material for stable isotope analysis. One species of amphipod, *Hyale plumulosa*, was identified to species. Invertebrates were washed, frozen, and stored and later combined into composite samples of at least 0.2 mg dry weight (i.e. many individuals comprised each sample). Pooling samples was necessary due to the small size/biomass of most of the invertebrates. When pooled samples were used, variance is reported as the variance across pooled samples.

Chum salmon fry typically migrate downstream to estuaries and nearshore marine habitats where they spend up to three weeks before making the transition to pelagic oceanic conditions [33]. Chum salmon fry are found in Howe Sound and the Strait of Georgia from March until late July and originate from the Squamish, Fraser, and other rivers discharging into the Strait [34]. Juvenile chum salmon were collected from March to June 2002 by beach seining at high tide using a 3 m×1 m beach seine with a mesh size of 6 mm set parallel to shore ∼1–3 m from the beach depending on the slope. Seining was conducted when the tide was higher than 3.05 m.

Chum salmon fry were kept in plastic bags in a cooler in the field and immediately frozen in the laboratory at −20°C. Fork length and wet weight were measured for 163 individual chum salmon fry and stomachs were removed from 28 fish for gut content analysis. Flank muscle tissue was then removed from 163 fish for stable isotope analysis. Fish samples for stable isotope analysis consisted of 1, 2 or 3 individuals. In total, stable isotope analysis was performed on 44 fish samples composed of 163 individual chum salmon fry. We have previously reported that there is no statistically significant difference in isotope values for fish samples composed of either individual fish or combined samples [12].

All samples were oven dried at 60°C until constant weight. Samples were then sent to the University of New Brunswick Stable Isotope Laboratory or to University of California at Davis Stable Isotope Laboratory where they were ground into powder. Samples of algae, supralittoral vegetation, invertebrates, and fish were oxidized, and the resulting CO_2_ and N_2_ were analyzed with a continuous flow-isotope ratio mass spectrometer. Ratios of carbon (^13^C/^12^C) and nitrogen (^15^N/^14^N) were expressed as the relative per mil (‰) difference between the sample and conventional standards (Pee Dee Belemite carbonate and N2 in air) as follows: ΔX = [R_sample_/R_standard_−1]×1000(‰), where X = ^13^C or ^15^N, and R = ^13^C:^12^C or ^15^N:^14^N.

Gut content analysis (GCA) was performed on 28 chum salmon fry. Gut contents were identified to lowest possible taxonomic level and results are shown for fraction of all individuals (numerical abundance summed over the 28 fish) and fraction occurrence (number of chum salmon fry with the prey item).

### Data analysis

Carbon and nitrogen isotope ratios were averaged across all sampling dates and the two sites. Contributions of terrestrially derived carbon (TC) and marine derived carbon (MC) to the assimilated carbon in chum salmon fry were calculated using the procedures and programs outlined in [32]. The mixing model calculates the contribution of each primary source assuming that only two sources are contributing to the isotopic signatures of the consumers. Source A was calculated as the average δ^13^C of supralittoral vegetation and source B was calculated as the average δ^13^C of marine macroalgae. For each taxa we report the δ^13^C and δ^15^N, relative proportion of TC, the standard error (SE) associated with the proportion, and the lower and upper 95%ile confidence intervals when n is = or >3. When n = 1 or 2 we only report δ^13^C and δ^15^N and relative proportion of TC. We were not able to use a three source mixing model using wrack detritus or POM because their isotopic signatures overlapped with either supralittoral vegetation or marine macroalgae (T. Romanuk, unpublished data; for a discussion of carbon sources in Howe Sound see [12]). The mixing model uses the same set of terrestrial and marine basal sources to calculate the relative proportions of terrestrial and marine carbon in the muscle tissue of chum salmon fry, thus the proportions of TC are qualitatively the same as those reported for δ^13^C.

This research was conducted according to relevant national guidelines of the Department of Fisheries and Oceans (Canada).

## Results

### Stable isotope analysis of food web components

#### δ^13^C and δ^15^N of primary producers and invertebrates

δ^13^C and δ^15^N of macroalgae was enriched and isotopically distinct from terrestrial vegetation. The average δ^13^C value for terrestrial vegetation was −28.34 (±2.43 SD; Table 1). The average δ^13^C value for marine macroalgae algae was −16.0 (±3.02 SD; Table 1).

**Table 1:** Stable isotope values of carbon (δ^13^C) and nitrogen (δ^15^N) for primary producers.

Habitat	Trophic Group	Common Name	Species	n	δ^13^C	δ^15^N
Supralittoral	vegetation	Red Alder	*Alnus rubra*	1	−28.78	−0.72
Supralittoral	vegetation	Salmonberry	*Rubus spectabilis*	1	−30.44	−0.5
Supralittoral	vegetation	Nootka Rose	*Rosa nutkana*	1	−27.77	−0.09
Supralittoral	vegetation	Grass	Poaceae	1	−30.04	0.64
Supralittoral	vegetation	Beach Pea	*Lathyrus japonicus*	1	−28.23	−0.44
Supralittoral	vegetation	Bracket Fungus		1	−22.71	−4.2
Supralittoral	vegetation	Western Red Cedar	*Thuja plicata*	1	−26.28	−3.53
Supralittoral	vegetation	Salal	*Gaultheria shallon*	1	−28.46	−3.16
Supralittoral	vegetation	Sitka Spruce	*Picea sitchensis*	1	−28.21	−1.81
Supralittoral	vegetation	Hairy Cat's Ear	*Hypochaeris radicata*	1	−31.69	−0.97
Supralittoral	vegetation	Black Twinberry	*Lonicera involucrata*	1	−27.37	−2.84
Supralittoral	vegetation	Blueberry	*Vaccinium* spp.	1	−31.81	−4.27
Supralittoral	vegetation	Moss	Bryophyta	1	−26.62	−0.34
Intertidal	macroalgae	Japanese Weed	*Sargassum muticum*	1	−14.64	2.83
Intertidal	macroalgae	Bleach Weed	*Prionitis lanceolatus*	1	−16.88	6.39
Intertidal	macroalgae	Black Tassel	*Pterosiphonia bipinnata*	1	−19.49	4.67
Intertidal	macroalgae	Tangle	*Laminaria* spp.	1	−10.83	6.84
Intertidal	macroalgae	Green Tuft	*Cladophora microcladioides*	1	−18.05	5.06
Intertidal	macroalgae	Rock Weed	*Fucus gardneri*	1	−16.12	4.47

Terrestrial vegetation was collected in the supralittoral and macroalgae was collected in the intertidal. Shown are common names and species names, number of samples (n), and sample δ^13^C and δ^15^N.

**Figure 3:**
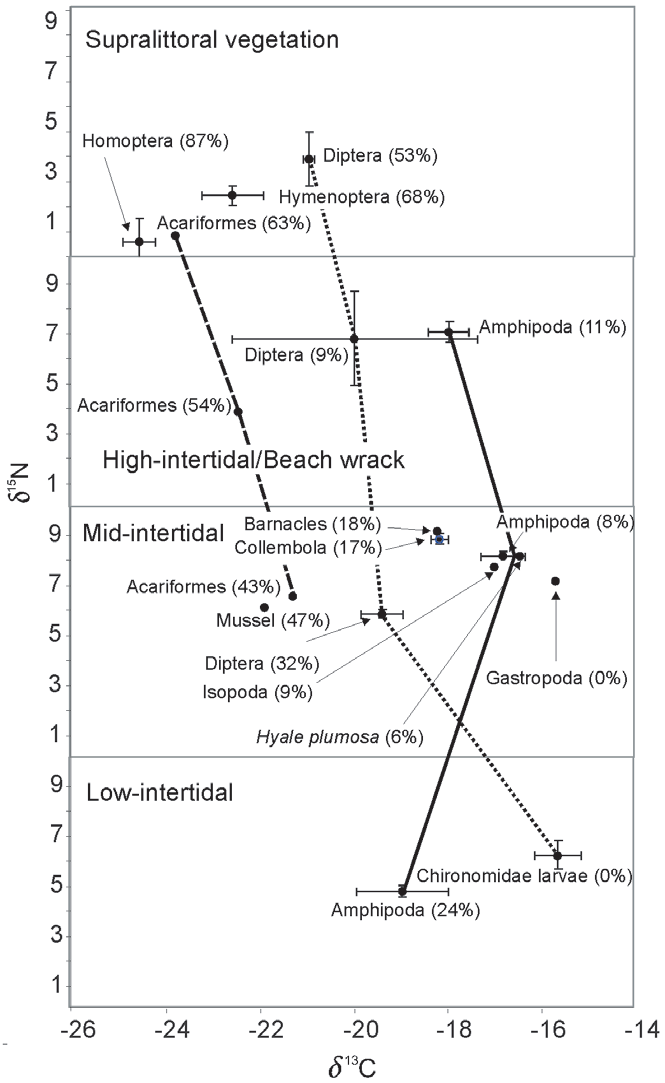
Carbon (δ^13^C) and nitrogen (δ^15^N) values of prey taxa in supralittoral, beach wrack, mid-intertidal and low-intertidal zones. Lines show taxa collected in more than two zones: Acariformes (hatched line), Diptera (dotted line), and Amphipoda (solid line). Values in brackets represent the fraction of terrestrially derived carbon (TC).

Mean δ^13^C and TC in invertebrates increased with elevation along the tidal gradient ranging from −17.28 (TC = 12%) in the low-intertidal to −18.43 (TC = 20%) in the mid-intertidal, −19.1(TC = 25%) in the high-intertidal, and −24.38 (TC = 68%) in the supralittoral. TC ranged from 0% (for low-intertidal chironomids and mid-intertidal gastropods) to 87.2% for supralittoral Homoptera (Table 2). No taxa had δ^13^C indicative of a 100% terrestrial carbon source and for some consumers enrichment increased toward the lower elevations. Of the three taxa present in more than three tidal zones, Dipteran and Acariformes showed a clear gradient of enrichment in δ^13^C and TC from the supralittoral zone to the low-intertidal zone (Fig. 3). In contrast, there was no clear pattern of enrichment in δ^13^C for Amphipoda from the high- to low-intertidal zones.

**Table 2:** Stable isotope values of carbon (δ^13^C) and nitrogen (δ^15^N) and proportion of terrestrially derived carbon (TC) for consumers (invertebrates, fish).

Habitat	Common Name/Taxa	n	Mean δ^13^C	Mean δ^15^N	SD δ^13^C	SD δ^15^N	% TC	SE TC	L 95%CI TC	U 95%CI TC
Low-intertidal	Amphipoda	6	−18.94	4.8	2	0.22	23.99	8.39	4.1	43.8
Mid-intertidal	Chironomidae	2	−15.63	6.26	0.51	0.58	0			
Mid-intertidal	Talitridae	3	−17	7.89	0.56	0.26	8.1	9.58	0	32.74
Mid-intertidal	Amphipoda *(Hyale plumulosa)*	3	−16.73	8.25	0.04	0.2	5.92	9.44	0	30.2
Mid-intertidal	Diptera	3	−19.96	9.45	3.04	2.63	32.09	15.82	0	82.5
Mid-intertidal	Mussels *(Mytilus sp.)*	1	−21.86	6.13			47.46			
Mid-intertidal	Gastropoda	1	−15.68	7.24			0			
Mid-intertidal	Barnacles	1	−18.17	9.14			17.59			
Mid-intertidal	Collembola	3	−18.15	8.85	0.19	0.21	17.42	8.38	0	39
Mid-intertidal	Acariformes	1	−21.29	6.55			42.79			
High-intertidal	Isopoda	1	−16.99	7.72			7.94			
High-intertidal	Talitridae	3	−17.4	7.24	0.48	0.58	11.35	9.18	0	34.9
High-intertidal	Diptera	3	−17.14	8.29	0.52	0.25	9.24	9.43	0	33.5
Supralittoral	Acariformes	1	−22.71	3.69			54.29			
Supralittoral	Diptera	3	−22.56	5.22	0.15	1.49	53.16	5.56	40.6	65.7
Supralittoral	Homoptera	3	−26.76	0.59	0.4	1.32	87.2	5.27	76	98.4
Supralittoral	Hymenoptera	3	−24.44	3.17	0.76	0.57	68.4	6.06	55	81.7
Supralittoral	Acariformes	1	−23.78	0.83			62.97			
Marine	Chum salmon fry	n = 44	−19.71	13.94	1.21	1.34	30.03	0.07	0.12	0.48
	*(Oncorhynchus keta)*	min	−23.59	10.4			12.78			
		max	−17.58	15.99			61.5			

Shown are values for taxa by habitat (supralittoral, high-intertidal, mid-intertidal, low-intertidal) and common name/taxa and species name. Shown are the number of samples(n), the mean and standard deviation (SD) for δ^13^C and δ^15^N, the proportion of TC (%) including the mean, standard error (SE), and upper (U) and lower (L) 95 percentile confidence limits of TC calculated using the mixing model (Phillips and Gregg 2001). For chum salmon fry the minimum and maximum values of δ^13^C, δ^15^N, and TC are also shown.

Mean δ^15^N was lowest in the supralittoral (2.45) and highest in the mid-intertidal (7.9) with low-intertidal (5.53) and high-intertidal (6.4) displaying intermediate values. δ^15^N for secondary consumers ranged from 0.59 to 9.45 (mean 6.18±2.67 SD; Table 2). Intertidal Diptera had the highest δ^15^N (9.45) followed by barnacles (9.14) and Collembola (8.85). Supralittoral Homoptera (0.59) and supralittoral Acariformes (0.83) had the lowest δ^15^N. The only taxa to show a trend in δ^15^N along the tidal gradient was Acariformes, with δ^15^N lowest in the supralittoral (0.83) and highest in the mid-intertidal (6.55; Fig. 3).

#### δ^13^C and δ^15^N of chum salmon fry

**Figure 4:**
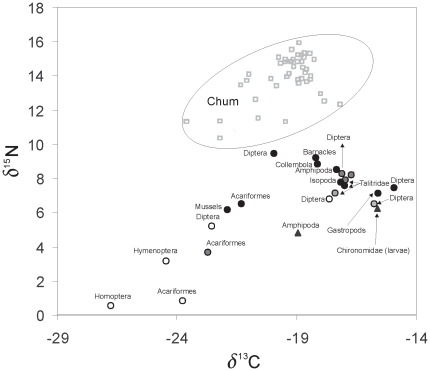
Carbon (δ^13^C) and nitrogen (δ^15^N) values for chum salmon fry and all prey taxa samples collected in the study. Habitat associations for the potential prey taxa are denoted by shaded circles or triangles: 1) white circles = supralittoral, 2) grey circles = high-intertidal/beach wrack, 3) black circles = mid-intertidal and 4) black triangles = low-intertidal. Chum salmon fry = open squares.

Chum salmon fry had an average fork length of 37 mm (range 29 to 52 mm) and an average wet weight of 0.48 g (range 0.2 to 1.35 g). δ^13^C for chum salmon fry averaged −19.71 (n = 44) ranging from −23.59 to −17.58 (±1.21 SD; Table 2 and Fig. 4) and δ^15^N averaged 13.94 ranging from 10.4 to 15.99 (±1.34 SD). TC ranged from 12.8 to 61.5% (mean 30%) with lower and upper confidence intervals of 12 and 48% (± SE 0.07).

#### Gut contents

Twenty-six prey taxa were identified in the gut content analysis of 28 individual chum salmon fry (Table 3). The five most abundant prey taxa by fraction of individual prey items were adult Chironomidae (60%), Harpacticoidea (8.9%), pupal Chironomidae (7.7%) gammarid Amphipoda (6.2), and larval Chironomidae (5%). Adult Chironomidae were present in 68% of individual chum followed by larval Chironomidae (50%), pupal Chironomidae (43%), gammarid Amphipoda (25%), *Corophium* sp. (Amphipoda; 21%), and Harpacticoidea (21%).

**Table 3:** Gut contents by fraction of total individuals and fraction of occurrence for 28 chum salmon fry.

Taxa		Life stage	Fraction of Individuals	Fraction of occurrence
Diptera	Chironomidae	adult	68.04%	17.46%
Diptera	Chironomidae	pupa	7.48%	7.94%
Oligochaeta	adult	5.61%	4.76%	
Amphipoda	Gammaridae	adult	4.30%	7.94%
Amphipoda	Corophiidae	adult	2.24%	7.94%
Teleost			1.87%	3.17%
Copepoda	Harpacticoida	adult	1.68%	4.76%
Cirripedia		larva	1.50%	6.35%
Diptera	Chironomidae	larva	1.50%	9.52%
Homoptera	Aphididae	adult	1.12%	6.35%
Amphipoda	Talitridae	adult	0.93%	1.59%
Cumacea		adult	0.75%	3.17%
Diptera	Ceratopogonidae	adult	0.56%	1.59%
Diptera	Ephydridae	adult	0.37%	1.59%
Diptera	Heleomyzidae	adult	0.37%	1.59%
Chelifera	Tanaidacea	adult	0.19%	1.59%
Plecoptera	Capniidae	adult	0.19%	1.59%
Coleoptera	Staphylinidae	larva	0.19%	1.59%
Diptera	Unidentified	larva	0.19%	1.59%
Diptera	Unidentified	adult	0.19%	1.59%
Diptera	Empididae	adult	0.19%	1.59%
Diptera	Sciaridae	adult	0.19%	1.59%
Hymenoptera	Eulophidae	adult	0.19%	1.59%
Arachnida	Araneae	adult	0.19%	1.59%
		n	728 prey items	28 fish

Shown is the taxa and life stage.

## Discussion

Our results suggest the importance of reciprocal subsidies in the terrestrial-marine ecotone in the Howe Sound estuary. Not only was marine derived carbon present in consumers present in the supralittoral zone, no supralittoral consumers were characterized by 100% terrestrially derived carbon. Likewise, terrestrially derived carbon was present even in the low-intertidal zone, particularly in amphipods. We found a clear gradient in terrestrially derived carbon down the tidal zone ranging from 68% across all taxa in the supralittoral to 25% in the high-intertidal, 20% in the mid-intertidal, and 12% in the low intertidal. This gradient was particularly clear for Diptera and Acariformes, two of the three taxa that were present in four or three zones respectively. In contrast to our results for carbon, there was no general spatial trend for δ^15^N suggesting that trophic position does not change systematically along the tidal gradient.

Stable isotope values of chum salmon fry and their prey indicated carbon contributions from both terrestrial and marine sources, with terrestrially derived carbon ranging from 12.8 to 61.5% in the muscle tissue of chum salmon fry (mean 30%). Adult chironomids were the dominant prey item of juvenile chum as has been previously reported at beaches in Howe Sound for juvenile chum salmon [15]. Stable isotope analysis of carbon in the intertidal Dipterans showed that between 9 and 53% of the carbon was terrestrially derived. Together, these results suggest that Dipterans are a major food web pathway for terrestrial carbon in chum salmon fry.

McCutchan et al. [35] has shown that enrichment of δ^13^C averages +0.4±0.12‰ (mean ± SE) from diet to consumer and δ^15^N averages +2.0±0.20‰ (mean ± SE) from diet to consumer. [35]. Our results suggest that: 1) adult Dipteran collected in the low and mid-intertidal, 2) Collembola and Amphipoda collected in the mid-intertidal, and 3) the amphipod *H. plumulosa* collected in the high-intertidal are the only groups of prey taxa that fall within potential δ^13^C and δ^15^N ranges for being a primary prey source (Fig. 4).

This interpretation is supported by the chum salmon fry gut content analysis, which found the highest number of individuals and highest occurrence of prey taxa in stomachs were adult, larval, and pupal Chironomidae. Collembola and Amphipoda were also abundant and common as food items. While the results from the stable isotope analysis also suggest that Cirripedia may be a primary prey source for chum salmon fry, the Cirripedia collected for stable isotope analysis were adults which may differ in their isotope ratios from free-living juveniles which are potential chum fry food. Six percent of fish had juvenile barnacles in the stomach contents, although the abundance of this prey item in the stomach contents was low (∼1%).

Taxa that fall outside of the above range of δ^15^N values may still be an important link [36] through either another consumer or because their basal source was significantly different from the basal source for chum (Fig. 4). These taxa include: 1) Acariformes collected from both the high- and mid-intertidal, 2) supralittoral Diptera, 3) Amphipoda collected from the high- and mid-intertidal, and 4) *Mytilus* sp. and Isopoda collected from the mid-intertidal (Fig. 4). All of these taxa except for *Mytilus* sp. larvae, the only life stage of *Mytilus* sp. that can be eaten by juvenile salmonids, were found in the gut contents (Table 3).

While the remaining groups fall outside the potential ranges for δ^13^C fractionation from diet to consumer [35]–[36], these taxa may still make up a portion of the diet of chum; however, their contribution to the isotopic values of chum is either marginal, or alternatively, opportunistic feeding on taxa with both strong terrestrial signatures such as Homoptera as well as taxa with strong marine signatures such as larval Chironomidae may have resulted in isotopic signatures that reflect a wide range of prey sources. For example, across all chum salmon fry analyzed we found that Homoptera made up 0.3% and larval Chironomidae made up 8.8% of the gut contents by number of individuals (Table 3).

In conclusion, our results show a clear gradient in the proportion of terrestrially derived carbon in invertebrate taxa that decreases down the tidal zone from 68% in the supralittoral to 25% in the high-intertidal, 20% in the mid-intertidal, and 12% in the low intertidal. Stable isotope values and gut content analysis of chum salmon fry indicated carbon contributions from both terrestrial and marine derived sources. Our results suggest that the vegetated supralittoral is an important trophic link in supplying terrestrial carbon to nearshore food webs.
